# From Slump to Comeback: Psychological Determinants of Performance Decline, Burnout, and Recovery in Competitive Athletes—A Systematic Review

**DOI:** 10.3390/sports14050165

**Published:** 2026-04-22

**Authors:** Yajuvendra Singh Rajpoot, Prashant Kumar Choudhary, Suchishrava Choudhary, Vasile-Cătălin Ciocan, Sohom Saha, Constantin Șufaru, Voinea Nicolae Lucian, Sema Arslan Kabasakal, Cristuta Alina Mihaela, Mihai Adrian Sava, Silviu-Ioan Pavel, Jolita Vveinhardt

**Affiliations:** 1 Department of Sports Management & Coaching, Lakshmibai National Institute of Physical Education, Gwalior 474002, India; yajupitu25@gmail.com; 2 Department of Physical Education Pedagogy, Lakshmibai National Institute of Physical Education, Gwalior 474002, India; prashantlnipe2014@gmail.com (P.K.C.); suchishrava05@gmail.com (S.C.); 3 Faculty of Movement, Sports, and Health Sciences, “Vasile Alecsandri” University of Bacău, 600115 Bacău, Romania; sufaruconstantin@ub.ro (C.Ș.); lucian.voinea@ub.ro (V.N.L.); cristuta.alina@ub.ro (C.A.M.); sava.adrian@ub.ro (M.A.S.); silviu.pavel@ub.ro (S.-I.P.); 4 Department of Sport Psychology, Lakshmibai National Institute of Physical Education, Gwalior 474002, India; sohomsaha77@gmail.com; 5 Department of Sports Health Sciences, Yalova University Faculty of Sports Sciences, Yalova 77000, Türkiye; sema.kabasakal@yalova.edu.tr; 6 Institute of Sport Science and Innovations, Lithuanian Sports University, Sporto St. 6, LT-44221 Kaunas, Lithuania

**Keywords:** psychological readiness, performance slump, burnout, return to sport, reinjury risk, athlete performance decline

## Abstract

Background: Psychological determinants are increasingly recognized as central contributors to both performance decline and recovery in competitive sport; however, contemporary evidence integrating injury-related and non-injury performance contexts remains fragmented. Objective: This systematic review synthesized empirical evidence (2016–2025) examining psychological determinants associated with return to sport (RTS), reinjury risk, burnout, injury incidence, and performance decline among competitive athletes. Methods: Conducted in accordance with PRISMA 2020 guidelines, a systematic search of PubMed, Scopus, Web of Science, and SPORTDiscus identified peer-reviewed studies published between January 2016 and December 2025. Eligibility criteria were defined using a PICO framework. Prospective cohort studies, longitudinal multi-wave investigations, one randomized controlled trial, matched cohort studies, diary-based designs, and injury-related observational studies were included. Due to heterogeneity in constructs and outcomes, findings were synthesized narratively. Results: Fourteen studies met the inclusion criteria, including prospective cohort studies, multi-wave longitudinal designs, one randomized controlled trial, one matched cohort study, and a diary-based investigation. Seven independent cohorts examined psychological readiness using the Anterior Cruciate Ligament—Return to Sport after Injury scale (ACL-RSI) in athletes with anterior cruciate ligament (ACL) injuries (sample sizes ranging from *n* = 39 to *n* = 384), consistently demonstrating that higher readiness predicted successful RTS at 6–24 months, while two prospective studies reported contrasting associations with second ACL injury risk. Four longitudinal studies (*n* = 93–491) showed that increased burnout and controlled motivation predicted performance decline and dropout trajectories, whereas higher resilience and mental toughness reduced burnout progression. One seasonal longitudinal study (*n* = 21) linked elevated cognitive anxiety and mood disturbance to increased injury incidence. Conclusion: Psychological determinants operate across deterioration and restoration pathways. Psychological readiness shows the strongest predictive consistency for RTS, while burnout, motivational climate, and resilience significantly shape long-term performance sustainability and injury-related outcomes.

## 1. Introduction

Performance in competitive sport is a multidimensional construct shaped not only by physiological and technical capacities but also by psychological mechanisms such as resilience, self-efficacy, and emotional regulation that influence adaptation to stress and adversity [1,2]. Contemporary sport psychology research emphasises that performance deterioration and recovery are not merely biomechanical or metabolic phenomena but are embedded within dynamic psychological processes governing motivation, resilience, emotional regulation, and cognitive appraisal that influence how athletes respond to pressure and rebound from setbacks [3,4]. Methodological advancements in evidence synthesis, including structured systematic approaches outlined in the Cochrane Handbook and PRISMA frameworks, have further reinforced the need to examine psychological determinants using rigorous longitudinal and interventional designs [5,6].

Performance slumps/decline, defined as sustained periods of underperformance relative to an athlete’s established competitive baseline, have been consistently linked to maladaptive psychological profiles. Recent systematic evidence further clarifies that performance decline represents a multidimensional phenomenon characterised by cognitive disruption, emotional dysregulation, loss of confidence, attentional narrowing, and maladaptive coping responses rather than purely technical or physical decline [7]. This synthesis indicates that performance decline is frequently preceded by elevated stress exposure, motivational conflict, and emerging burnout symptoms, reinforcing the need to conceptualize performance deterioration within a broader biopsychological framework. Longitudinal investigations demonstrate that perfectionistic concerns predict burnout trajectories over time, with motivational regulation mediating this association [8]. Complementary evidence indicates that mental toughness may attenuate burnout symptoms, though its protective influence appears context-dependent [9]. More recent systematic reviews and meta-analyses extend this evidence by demonstrating that mental toughness is positively associated with competitive performance outcomes and negatively associated with psychological distress [10]. In addition, structured mental toughness training interventions produce small-to-moderate improvements in resilience, coping effectiveness, and performance-relevant indicators across sport levels, suggesting that mental toughness represents a modifiable psychological resource rather than a static dispositional trait [11]. Foundational work on mental toughness further demonstrates associations with motivational antecedents and psychological health, suggesting that adaptive psychological profiles may buffer against performance instability [12,13].

Conversely, controlled motivation and exposure to controlling coaching behaviours have been prospectively associated with increased ill-being and maladaptive growth trajectories across competitive seasons [14,15]. A comprehensive systematic review and meta-analysis further demonstrates that coach autonomy-supportive behaviours are positively associated with intrinsic motivation, well-being, and sustained sport engagement, whereas controlling coaching climates are linked to ill-being and maladaptive motivational regulation [16]. Grounded in self-determination theory, these findings reinforce the ecological influence of coaching climate on performance sustainability and psychological health across competitive levels.

These findings collectively highlight the interactive role of individual dispositions and environmental climate in shaping performance vulnerability. High-level syntheses reinforce these longitudinal findings. A systematic review of factors associated with athlete burnout in team sports identified perfectionistic concerns, controlling coaching behaviours, excessive training demands, and low autonomy support as consistent correlates of burnout symptomatology [17]. Complementing this, a recent systematic review and meta-analysis examining mental and physical health outcomes of athlete burnout reported significant associations with depressive symptoms, anxiety, sleep disturbance, and reduced physical well-being [18]. Moreover, cross-temporal meta-analytic evidence indicates that average burnout symptom levels have increased between 1997 and 2019, suggesting that burnout represents an escalating challenge within contemporary competitive sport environments [19].

Protective psychological constructs such as resilience and optimism have emerged as central mechanisms in buffering against both performance decline and dropout. Longitudinal person-oriented research demonstrates that resilience mitigates sport and school burnout risk among student-athletes [20]. Resilience refers to the ability to adapt positively to stress, adversity, or performance challenges while maintaining or regaining psychological well-being and functional performance. Correlational and applied studies further associate resilience and optimism with enhanced competitive performance outcomes, and systematic evidence indicates that higher resilience relates to better athletic performance and recovery following injury. Recent research also suggests that resilience facilitates recovery via emotion regulation and self-efficacy pathways, with psychological resilience and emotional regulation contributing to stronger self-efficacy beliefs that, in turn, support rehabilitation success. In applied contexts, expert athletes overcoming performance decline report psychological resilience as a core adaptive resource [21]. Although mindfulness-based interventions have demonstrated benefits for psychological well-being and anxiety regulation in athletes, none of the studies included in the present review directly examined mindfulness as a primary determinant of performance decline or recovery outcomes. Therefore, its role within this synthesis remains indirect and highlights an important gap for future research [22]. An umbrella review synthesizing multiple systematic reviews further concludes that mindfulness-based programmes show consistent benefits for stress reduction and mental health, although variability in intervention structure and outcome measurement remains substantial [23]. These findings position emotion regulation and mindfulness as potentially scalable mechanisms for both performance stabilisation and psychological recovery.

Injury-related performance disruption represents a particularly critical domain in which psychological determinants exert measurable influence. Psychological readiness to return to sport (RTS), commonly assessed using the ACL-RSI scale, has demonstrated predictive validity for successful RTS and second-injury risk following anterior cruciate ligament reconstruction [24,25]. Psychological readiness to return to sport refers to an athlete’s cognitive, emotional, and confidence-based preparedness to resume sport participation following injury. Recent higher-level syntheses further strengthen this evidence base. A systematic review with meta-analysis examining ACL-RSI trajectories over time demonstrates that psychological readiness scores generally increase from early rehabilitation to 12–24 months post-reconstruction and are consistently associated with successful return to preinjury sport levels [26]. Complementing this, a systematic review and meta-analysis of predictors of return to sport and reinjury after ACL reconstruction identifies psychological readiness, fear of reinjury, and self-efficacy as significant contributors alongside physical performance criteria [27]. Moreover, network meta-analytic evidence integrating psychological and physical return-to-sport criteria suggests that optimal RTS outcomes are achieved when multidimensional assessment frameworks are used rather than isolated biomechanical benchmarks [28].

Associations between readiness and biomechanical recovery indicators such as knee laxity have further underscored the multidimensional nature of rehabilitation outcomes [29]. Randomized clinical evidence indicates that surgical technique may indirectly influence psychological readiness trajectories, with bridge-enhanced ACL restoration demonstrating higher readiness scores at six months compared to autograft reconstruction [30]. Subgroup analyses confirm consistent readiness-RTS associations across injury variations [31], while subjective running ability and hop performance have been shown to influence ACL-RSI subdomains [32]. Beyond ACL injury, psychological trauma and posttraumatic stress symptomatology have been documented following ulnar collateral ligament injuries in baseball players [33], reinforcing the broader psychological burden of musculoskeletal injury.

Prospective injury-etiologic research further demonstrates that negative mood states and competitive anxiety predict subsequent injury occurrence in elite female volleyball players [34]. Diary-based methodologies reveal daily stress fluctuations influencing coping effectiveness among elite esports athletes, emphasising the temporal and dynamic character of psychological adaptation [35]. Recent meta-analytic evidence further supports a bidirectional relationship between mental health and sports injury. A systematic review and meta-analysis demonstrate that pre-existing psychological distress increases subsequent injury risk, while injury exposure is associated with elevated depressive and anxiety symptoms, indicating reciprocal vulnerability pathways [36]. Complementing this, meta-analytic findings suggest that structured psychological interventions targeting stress management, coping skills, and cognitive restructuring can significantly reduce injury incidence, highlighting the preventive potential of psychological training within high-performance sport contexts [37]. Prospective weekly monitoring research further illustrates this dynamic relationship, with sustained mental distress linked to increased injury and illness risk across competitive seasons [38]. Such findings align with COSMOS-E guidance, which stresses the importance of temporality, longitudinal modelling, and methodological rigour in etiological research [39]. Moreover, resilience has been associated with shorter recovery timelines following athletic injuries, supporting its integrative role across deterioration and recovery pathways.

Despite substantial advances, the literature remains fragmented across discrete domains of burnout, performance decline, injury incidence, and return-to-sport readiness without an integrative synthesis examining psychological determinants as both risk and protective mechanisms across the full performance continuum. Importantly, the current body of evidence appears disproportionately weighted toward injury-related return-to-sport (RTS) outcomes, particularly anterior cruciate ligament (ACL) populations, whereas comparatively fewer longitudinal investigations have examined psychological determinants of performance decline, burnout, and resilience in non-injury contexts. Many prior investigations employ isolated outcome measures or cross-sectional designs, limiting causal inference and temporal clarity. Furthermore, inconsistencies in reinjury prediction and heterogeneity in methodological quality underscore the need for structured synthesis guided by contemporary systematic review standards [5,40]. Rigorous appraisal frameworks such as RoB 2 for randomised trials, ROBINS-I for non-randomised studies [41], and the Newcastle-Ottawa Scale for observational research [42] provide necessary tools for evaluating bias and strengthening inferential validity.

To address the conceptual breadth of psychological determinants across performance and injury domains, the present review adopts a biopsychosocial performance framework. Rather than examining isolated constructs, this review conceptualizes psychological factors as operating across an integrated continuum encompassing performance decline, burnout progression, injury occurrence, return-to-sport transitions, and reinjury vulnerability. This approach is justified by emerging evidence indicating that shared psychological mechanisms, such as stress appraisal, motivation, resilience, and emotional regulation, underlie both deterioration and recovery processes in sport. Accordingly, the review does not treat these domains as independent outcomes but as interrelated components of athlete performance trajectories. To improve conceptual clarity, the present review focuses on integrating these constructs within a unified performance decline–recovery framework rather than treating them as isolated phenomena. Although injury-related outcomes may appear as a distinct domain, emerging evidence suggests that performance decline, injury occurrence, and return-to-sport processes are interconnected within a shared biopsychosocial framework. Psychological mechanisms such as stress appraisal, motivation, resilience, and emotional regulation operate across both performance and injury contexts, influencing vulnerability, adaptation, and recovery trajectories. Therefore, the inclusion of injury-related outcomes in the present review is conceptually justified as part of an integrated performance decline–recovery continuum rather than as a separate line of inquiry.

Therefore, the objective of this systematic review was to synthesize prospective, longitudinal, and experimental evidence (2016–2025) examining how psychological determinants influence performance decline, burnout, recovery, and return-to-sport outcomes among competitive athletes. By integrating etiological and interventional evidence within a structured methodological framework consistent with PRISMA 2020 and Cochrane recommendations [5,43], this review aims to provide a comprehensive conceptual model linking psychological adaptation processes to measurable performance outcomes across the athletic lifespan.

## 2. Materials and Methods

### 2.1. Study Selection Procedures

**Figure 1:**
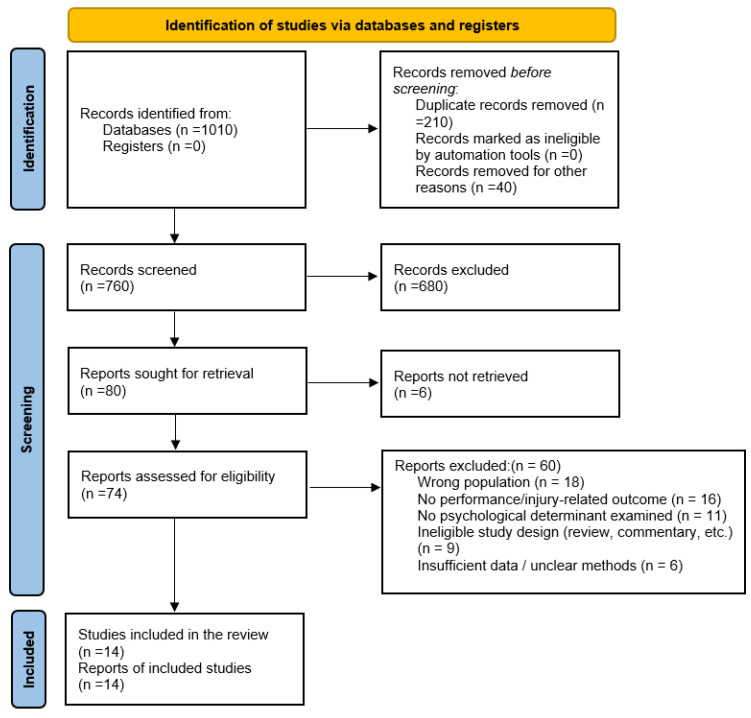
PRISMA 2020 Flow Diagram of Study Selection Process.

The protocol for this systematic review was prospectively registered in the PROSPERO International Prospective Register of Systematic Reviews (CRD420261342274; https://www.crd.york.ac.uk/PROSPERO/view/CRD420261342274, accessed on 16 March 2026). This systematic review was conducted in accordance with the Preferred Reporting Items for Systematic Reviews and Meta-Analyses (PRISMA 2020) (See Figure 1) guidelines (PRISMA Statement), which provide updated standards for transparent reporting of systematic reviews [43]. The completed PRISMA checklist is provided in the Supplementary File S1. Eligibility criteria were established a priori using a PICO framework (See Table 1), consistent with contemporary evidence synthesis methodology recommendations [5,43]. Studies were included if they involved competitive athletes (youth, collegiate, elite, or professional), examined psychological determinants (e.g., resilience, mental toughness, burnout, motivation, psychological readiness, stress, coping, anxiety, PTSD symptomatology), and evaluated performance-related outcomes such as return to sport (RTS), reinjury risk, burnout, injury incidence, performance decline, or psychological adaptation. Eligible designs included prospective cohort studies, longitudinal investigations (≥2 time points), randomized controlled trials, matched cohort studies, diary-based temporal studies, and injury-related observational studies examining psychological responses to injury or performance decline, in line with methodological guidance for synthesizing observational and interventional sport science research [5,39]. Studies were excluded if they were reviews, meta-analyses, scoping reviews, consensus statements, dissertations, conference abstracts, or involved non-athlete populations. Two reviewers independently screened titles and abstracts, followed by full-text assessment, adhering to recommended duplicate screening procedures to minimize selection bias [5,43]. Disagreements were resolved through discussion until a consensus was reached.

**Table 1:** Inclusion and Exclusion Criteria.

PICO Element	Inclusion Criteria (STRICT)	Exclusion Criteria (STRICT)
P—Population	Competitive athletes (youth, collegiate, elite, professional) and post-injury athletes (e.g., ACL, UCL, concussion) actively engaged in sport	Non-athletes, recreational-only populations, general population, clinical non-sport samples
I/E—Exposure (Psychological Determinants)	Psychological resilience, mental toughness, burnout, motivation, psychological readiness to return to sport (RTS), stress, coping, emotion regulation, competitive anxiety, PTSD symptoms, self-efficacy when linked to performance decline or injury recovery	General mental health prevalence studies without sport-performance or injury context; academic dual-career adjustment only; alcohol-only psychological studies without performance linkage
C—Comparator	Comparison across time (longitudinal follow-up), between psychological levels (high vs. low resilience/readiness), injury vs. non-injury groups, surgical technique comparisons, or matched cohorts	Studies without any comparator, predictor, or analytical relationship (purely descriptive reports)
O—Outcomes	Performance slump (burnout, performance decline), return-to-sport outcomes, reinjury risk, injury-related psychological distress, recovery trajectory, seasonal performance changes, perceived performance fluctuations	Injury incidence without psychological predictors; pure mental health epidemiology; motivation-only outcomes without performance consequences
Study Design (Methodological Filter)	Prospective cohort, longitudinal (≥2 time points), diary study, randomized controlled trial, matched cohort, and high-quality cross-sectional studies assessing psychological response to injury/performance decline	Systematic reviews, meta-analyses, scoping reviews, narrative reviews, consensus statements, policy papers, theoretical/conceptual papers, dissertations, conference abstracts
Publication Year	January 2016–December 2025	Before 2016 or after 2025
Language & Publication Type	English; peer-reviewed indexed journal articles	Non-English publications; books; book chapters; theses

### 2.2. Literature Search: Administration and Update

A comprehensive electronic search was conducted across major databases, including PubMed, Scopus, Web of Science, and SPORTDiscus. Although PsycINFO is a core database for psychological research, it was not included in the present search strategy due to substantial overlap with indexed records in Scopus and Web of Science. Additionally, SPORTDiscus was prioritized given its specific relevance to sport science and athlete populations. This decision may have limited the identification of some psychology-focused studies and is acknowledged as a methodological limitation. The search strategy combined keywords and Boolean operators related to: (“psychological resilience” OR “mental toughness” OR “burnout” OR “psychological readiness” OR “stress” OR “coping” OR “anxiety” OR “PTSD”) AND (“athlete” OR “sport”) AND (“performance slump” OR “return to sport” OR “injury recovery” OR “burnout” OR “reinjury”). Searches were limited to peer-reviewed articles published in English between January 2016 and December 2025. Reference lists of eligible studies were manually screened to identify additional relevant articles. The search was last updated prior to final manuscript submission to ensure inclusion of the most recent studies. The time restriction (2016–2025) was applied to ensure inclusion of contemporary evidence reflecting recent methodological advancements, updated psychological frameworks, and current sport performance environments. Earlier studies were excluded to minimize conceptual heterogeneity and enhance the relevance of findings to modern competitive sport contexts.

### 2.3. Data Extraction

Data were independently extracted using a standardized extraction form, consistent with recommended best practices for systematic reviews [5,43]. The following information was recorded: author(s), publication year, country, sport and athlete population, sample size, study design, psychological variables measured (including instruments used), outcome variables, follow-up duration, statistical analyses, and key findings. The use of structured extraction templates enhances transparency, reproducibility, and methodological rigour in evidence synthesis [5]. Clear documentation of extracted variables and analytical approaches is also recommended in reporting guidance for reviews of observational studies and narrative syntheses.

When necessary, corresponding authors were contacted for clarification of methodological details to minimize reporting bias and incomplete data [43]. Extracted data were cross-verified by the review team to ensure accuracy and completeness and to reduce extraction errors and reviewer bias [5].

### 2.4. Methodological Quality of the Included Studies

Methodological quality was assessed according to study design, following contemporary risk-of-bias evaluation standards in systematic reviews [5]. Prospective and cohort studies were evaluated using adapted Newcastle–Ottawa Scale criteria, which are commonly applied for assessing the quality of non-randomized studies in meta-analyses [42]. The randomized controlled trial was assessed using Cochrane risk-of-bias principles consistent with the updated RoB 2 framework [5,41]. Longitudinal and other observational study designs were evaluated for potential sources of bias and selective reporting [41]. Cross-sectional studies were appraised using criteria adapted from the Joanna Briggs Institute critical appraisal checklist. Risk of bias assessment was conducted independently by two reviewers. Discrepancies were resolved through discussion until consensus was reached, ensuring reliability and minimizing subjective bias. For each included study, domain-level assessments were conducted, and judgments were assigned as low, moderate, or high risk of bias based on predefined criteria aligned with the respective appraisal tools. Specifically, studies were classified as low risk when all domains demonstrated minimal bias, moderate risk when at least one domain indicated some concern without reaching high risk, and high risk when one or more domains demonstrated substantial methodological limitations. These assessments were performed independently and cross-verified to enhance reliability and reduce subjective bias. Methodological quality ratings were derived from domain-level evaluations to facilitate transparent interpretation of the strength and credibility of the synthesised evidence, consistent with recommended systematic review reporting standards [5,43].

### 2.5. Summary Measures

Primary summary measures included the direction and strength of association between psychological determinants and performance-related outcomes, consistent with recommendations for synthesizing both interventional and observational evidence [43]. For prospective and longitudinal studies, reported regression coefficients, odds ratios, mediation effects, and trajectory analyses were examined, as recommended for interpreting associations in cohort and etiological research [5,39]. For the randomized controlled trial, between-group differences and intervention effects were considered using reported effect estimates and confidence intervals, in line with Cochrane guidance for comparative effectiveness synthesis [5]. Due to methodological heterogeneity across psychological constructs, measurement instruments, and outcome variables, pooled effect sizes were not calculated. In such cases, narrative synthesis is recommended when statistical aggregation is inappropriate because of conceptual and statistical heterogeneity [6]. The synthesis, therefore, focused on consistency, magnitude, and theoretical coherence of reported associations across studies. To enhance interpretative clarity, a hierarchy of evidence was applied during synthesis. Randomized controlled trials were considered the highest level of evidence, followed by prospective cohort studies, longitudinal multi-wave designs, matched cohort studies, and diary-based investigations. Cross-sectional studies were interpreted with caution due to the absence of temporal sequencing. Conclusions were weighted accordingly, with greater emphasis placed on findings derived from prospective and experimental designs.

### 2.6. Synthesis of Results

Given the heterogeneity in study designs, psychological constructs, outcome measures, and statistical reporting, a meta-analysis was not conducted. Decisions regarding quantitative synthesis were made in accordance with methodological guidance recommending against statistical pooling when substantial clinical, methodological, or conceptual heterogeneity is present [5]. Instead, a structured narrative synthesis approach was employed. Studies were grouped according to primary outcome domains: (1) return to sport and reinjury risk, (2) burnout and performance decline, and (3) injury incidence and psychological adaptation. Findings were further interpreted based on study design hierarchy, with greater weight assigned to randomized controlled trials and prospective cohort studies. Patterns of consistency, direction of associations, and theoretical coherence were examined across studies. Where findings were heterogeneous or conflicting, differences in study design, population characteristics, and measurement approaches were considered. Evidence strength was interpreted based on methodological rigour, temporal design, and consistency of results across independent samples. This approach aligns with structured narrative synthesis frameworks that emphasize transparency in grouping studies, exploring patterns, and assessing the robustness of conclusions [5,6,40].

### 2.7. Publication Bias

Formal assessment of publication bias (e.g., funnel plot asymmetry or Egger’s regression test) was not performed due to the absence of pooled effect sizes and the substantial heterogeneity of study designs, psychological constructs, and outcome measures. Statistical methods for detecting small-study effects are generally recommended only when a sufficient number of comparable studies are included in a meta-analysis (typically ≥10) and when effect sizes are pooled [5]. Given the narrative synthesis approach adopted in the present review, formal quantitative assessment of publication bias was not methodologically appropriate [41,43]. However, inclusion of studies across multiple countries, sports, competitive levels, and methodological designs reduced the likelihood of systematic reporting bias and enhanced the breadth of evidence representation. Additionally, screening procedures and predefined eligibility criteria were implemented to minimize selective inclusion and enhance transparency [5].

### 2.8. Additional Analyses

Subgroup patterns were explored qualitatively across injury-related versus non-injury performance contexts. Differences in psychological determinants across sport types (e.g., individual vs. team sports, traditional sports vs. esports) were examined descriptively. Study design hierarchy was considered when interpreting evidence strength. Sensitivity considerations were applied to cross-sectional findings to avoid overinterpretation of non-temporal associations.

## 3. Results

The systematic search yielded 14 studies that met the predefined PICO-based eligibility criteria and were included in the final synthesis. The included studies comprised prospective cohort designs, longitudinal multi-wave investigations, one randomized controlled trial, a matched cohort study, an intensive diary study, and one cross-sectional injury-related investigation. Collectively, the evidence examined psychological determinants, including resilience, mental toughness, burnout, motivation, psychological readiness to return to sport (ACL-RSI), stress, coping, anxiety, and PTSD symptomatology. Outcome domains primarily focused on return-to-sport success, reinjury risk, performance decline, burnout trajectories, injury incidence, psychological adaptation, and slump recovery processes. Overall, the methodological quality was predominantly low-to-moderate risk of bias, with the strongest evidence emerging from prospective and randomized designs examining injury recovery and return-to-sport outcomes. The relatively small number of included studies (*n* = 14) reflects the application of strict inclusion criteria prioritizing prospective, longitudinal, and experimental designs. Many studies were excluded due to cross-sectional design, lack of performance-related outcomes, or absence of temporal data, highlighting a limited availability of high-quality longitudinal evidence in this research domain.

Table 2 presents the characteristics of the 14 studies that met the predefined inclusion criteria (2016–2025; longitudinal, prospective, retrospective, matched cohort, diary design, or randomized controlled trial).

The studies investigate psychological determinants such as resilience, mental toughness, burnout, stress, motivation, emotion regulation, and psychological readiness, and their associations with performance decline, burnout trajectories, recovery processes, return-to-sport outcomes, and reinjury risk among competitive athletes.

In Table 3 methodological quality across the included studies was predominantly low-to-moderate risk of bias. Prospective cohort studies and the randomized controlled trial demonstrated the strongest internal validity, with minimal concerns regarding selection, measurement, and reporting bias.

Longitudinal studies generally showed low selection bias but moderate concerns related to self-reported psychological measures and confounding control. Attrition bias was minimal across most studies, thanks to robust follow-up procedures.

In Table 4, across the included studies, burnout emerged as the most consistently supported psychological determinant of performance-related decline. Four longitudinal investigations demonstrated that elevated burnout symptoms were associated with increased dropout risk, reduced sport functioning, and progressive performance deterioration. Similarly, resilience and mental toughness were identified as protective factors, with higher levels predicting reduced burnout trajectories and improved adaptation over time. Within motivational constructs, controlled motivation and perceptions of controlling coaching behaviours were consistently associated with maladaptive outcomes, including increased ill-being and burnout progression.

Evidence related to stress appraisal and mood states was more limited, with diary and small-sample longitudinal designs suggesting that heightened stress intensity and cognitive anxiety may contribute to maladaptive coping patterns and elevated injury vulnerability. Psychological readiness to return to sport (ACL-RSI) demonstrated strong and consistent predictive value for return-to-sport outcomes across seven injury cohorts. However, findings regarding reinjury risk were inconsistent, with some studies indicating that lower readiness increased reinjury risk, while others suggested that higher readiness was associated with subsequent injury. Overall, the strongest evidence base supported burnout and psychological readiness as key determinants of performance-related outcomes, whereas stress and mood constructs showed emerging but less conclusive evidence. To avoid redundancy with Table 2 and Table 4, Table 5 provides a condensed integrative overview of key outcome domains.

Table 5 presents an integrated synthesis of the psychological determinants influencing key performance-related outcomes across the included studies, highlighting consistent patterns across return to sport (RTS), reinjury risk, burnout, injury incidence, and coping effectiveness. Strong evidence indicates that psychological readiness, particularly as measured by ACL-RSI and self-efficacy, is a central predictor of successful RTS, although its relationship with reinjury risk appears complex and, in some cases, paradoxical.

Longitudinal findings consistently demonstrate that maladaptive motivational climates, perfectionism, and controlling coaching behaviours elevate burnout trajectories, whereas resilience and mental toughness exert protective effects. Prospective data further reveal that heightened cognitive anxiety and mood disturbances are associated with increased injury incidence across competitive seasons. Overall, the synthesis underscores the multidimensional and bidirectional role of psychological factors in shaping both performance sustainability and health-related outcomes in athletes.

Given the limited number of included studies and heterogeneity in study designs, a formal certainty-of-evidence assessment was conducted using principles adapted from the GRADE framework. Overall, the certainty of evidence was rated as moderate for return-to-sport outcomes due to consistency across prospective cohorts, low-to-moderate for burnout and performance decline outcomes, and low for injury incidence and coping-related findings due to smaller sample sizes and observational designs. These ratings reflect the variability in methodological rigor, sample size, and consistency of findings across domains.

## 4. Discussion

The purpose of this systematic review was to synthesize contemporary empirical evidence (2016–2025) examining psychological determinants associated with performance decline and recovery among competitive athletes. The findings demonstrate that psychological factors operate across two interconnected pathways: (1) deterioration mechanisms contributing to burnout, injury risk, reinjury, and performance decline, and (2) protective or recovery-oriented mechanisms facilitating return to sport, adaptation, and restoration of performance. Overall, the consistent evidence emerged from prospective cohort and longitudinal designs, particularly within injury-related return-to-sport (RTS) contexts.

### 4.1. Psychological Readiness and Return to Sport

Psychological readiness, particularly as operationalised by the ACL-RSI scale, emerged as one of the most consistently supported determinants of successful return to sport and reinjury risk. Across multiple prospective cohort studies, higher psychological readiness predicted successful RTS outcomes [24]. Importantly, lower readiness scores were associated with increased risk of second ACL injury [25], indicating that psychological preparedness is not merely an adjunct factor but may influence reinjury vulnerability. Evidence from a randomized controlled trial further demonstrated that surgical technique influenced psychological readiness trajectories, indirectly affecting RTS outcomes [30]. Matched cohort findings similarly supported the relevance of readiness across injury subgroups [31]. Collectively, these findings suggest that psychological readiness functions as a key transitional determinant linking rehabilitation completion to performance reintegration. Although cross-sectional in nature, this finding underscores that injury-related psychological distress may meaningfully influence recovery trajectories. These results align with broader evidence indicating that psychological adaptation processes are integral to injury rehabilitation rather than secondary considerations. Notably, conflicting findings were observed regarding the relationship between psychological readiness and reinjury risk. While some studies indicated that lower readiness predicted reinjury, others reported that higher readiness was associated with increased reinjury incidence. This apparent contradiction may be explained by differences in return-to-sport timing, exposure levels, and athlete risk-taking behaviour. Athletes with higher psychological readiness may return to high-intensity competition earlier, thereby increasing exposure to reinjury risk, whereas those with lower readiness may delay return, reduce immediate risk but potentially reflect incomplete psychological recovery. Additionally, variations in measurement timing and sample characteristics may contribute to these inconsistencies. These findings highlight the need for multidimensional return-to-sport decision models integrating both psychological and physical readiness indicators.

### 4.2. Burnout, Motivation, and Performance Decline

In non-injury contexts, longitudinal evidence consistently linked maladaptive motivational and stress processes to burnout and performance deterioration. Madigan et al. (2016) demonstrated that motivation mediated the relationship between perfectionism and burnout across three waves, highlighting the importance of self-determined regulation in sustaining performance [8]. Similarly, Madigan and Nicholls (2017) found that mental toughness was inversely associated with burnout development over time. Environmental factors further influenced these trajectories [9]. Controlling coaching behaviours were associated with increased burnout levels across seasons [15], and controlled forms of motivation predicted ill-being progression in latent growth modelling analyses [14]. These findings suggest that burnout-related performance decline is not solely an intrapersonal phenomenon but is embedded within motivational climates and coaching dynamics. The multi-year longitudinal study by Sorkkila et al. (2019) provided additional insight, demonstrating that resilience functioned as a protective factor against burnout and dropout trajectories among student-athletes [20]. Taken together, this body of work supports a temporal pathway in which stress, maladaptive perfectionism, and controlling climates contribute to burnout, while resilience and mental toughness mitigate deterioration.

### 4.3. Resilience and Performance Recovery

Resilience emerged as a protective psychological factor primarily in relation to burnout and dropout trajectories rather than direct performance outcomes. Longitudinal evidence demonstrated that higher resilience was associated with reduced burnout progression and lower dropout risk among student-athletes [20]. These findings suggest that resilience may operate indirectly by sustaining psychological well-being and motivation over time, thereby supporting performance continuity rather than directly enhancing performance metrics. However, no included studies provided robust objective performance indicators (e.g., match statistics or ranking outcomes) linked directly to resilience, which limits conclusions regarding its direct performance effects. This gap highlights the need for future research integrating resilience measures with objective performance data to better understand its role in performance recovery and sustainability.

### 4.4. Stress, Anxiety, and Injury Incidence

Prospective findings also highlighted the role of mood disturbance and competitive anxiety in injury risk. Boladeras et al. (2025) reported that elevated anxiety and negative mood states predicted injury occurrence across a competitive season in elite female volleyball players [34]. These findings suggest that emotion regulation strategies may serve as protective mechanisms within high-pressure environments. Diary-based evidence further revealed that daily stress fluctuations influenced coping effectiveness in elite esports athletes [35], emphasizing the temporal and dynamic nature of psychological processes across micro-cycles of competition.

### 4.5. Integration of Deterioration and Restoration Pathways

Collectively, the reviewed studies indicate that psychological determinants function along a continuum of risk and protection. Burnout, controlled motivation, chronic stress, and anxiety were associated with performance decline, ill-being progression, and injury risk [8,34]. Conversely, resilience, mental toughness, adaptive emotion regulation, and psychological readiness were linked to recovery, adaptation, and successful return to sport [20]. The consistent evidence suggests base emerged in injury rehabilitation contexts, particularly ACL populations, where psychological readiness demonstrated consistent predictive value across independent cohorts [24,25]. Importantly, while many studies reported significant associations, causality cannot be definitively inferred due to the predominance of observational designs. Nevertheless, longitudinal and prospective methodologies enhance confidence in temporal sequencing compared to cross-sectional evidence. The overall pattern of findings suggests that psychological processes are integral components of both performance decline and restoration in sport.

Importantly, the present findings extend beyond descriptive synthesis by highlighting a unified explanatory framework in which psychological determinants operate across both deterioration and recovery processes. Rather than functioning as isolated predictors, constructs such as resilience, motivation, and stress appraisal appear to interact dynamically across time, shaping athlete trajectories in a non-linear manner. This reinforces the need for integrative models in sport psychology that account for both vulnerability and adaptation processes simultaneously. Future research should prioritize longitudinal and interventional designs to clarify causal mechanisms and to identify optimal psychological intervention strategies that can be implemented across different phases of athletic performance and recovery.

### 4.6. Practical and Research Implications

This review provides value to society by reinforcing that athlete performance and recovery are deeply influenced by psychological health, thereby supporting a more holistic model of sport development. For coaches, it highlights the importance of fostering autonomy-supportive climates, monitoring burnout indicators, and integrating psychological readiness assessments into routine training and rehabilitation processes. For athletes, it underscores that resilience, mental toughness, and emotional regulation are trainable competencies that can buffer performance decline and injury-related distress. Sports medicine professionals and rehabilitation teams can use these findings to incorporate structured psychological screening, particularly during return-to-sport decision-making. Sporting organizations and governing bodies may benefit by embedding mental performance frameworks into talent development pathways to reduce dropout and reinjury risk. From an industry perspective, the evidence supports investment in sport psychology services, athlete monitoring technologies, and resilience-based training programs. Collectively, the review advances a performance model where psychological determinants are recognized as central, rather than secondary, to sustainable athletic success and well-being.

### 4.7. Limitations

This review has a few limitations. Most included studies were observational, limiting causal inference despite longitudinal designs. Considerable heterogeneity in psychological constructs, measurement tools, sport contexts, and outcome definitions prevented meta-analysis and may affect comparability. The predominance of self-report measures introduces potential measurement and reporting bias. Additionally, restriction to English-language peer-reviewed publications and limited interventional evidence may reduce generalizability and increase the risk of publication bias.

## 5. Conclusions

This systematic review synthesises contemporary evidence demonstrating that psychological determinants play a central role in both performance decline and recovery among competitive athletes. Psychological readiness emerged as one of the most consistently supported determinants of return-to-sport success and reinjury risk, particularly in injury contexts. Burnout, stress, and controlling motivational climates were longitudinally associated with performance deterioration, whereas resilience and mental toughness functioned as protective mechanisms. Injury-related psychological distress, including PTSD symptomatology, further underscores the emotional impact of sport trauma. Overall, the findings support an integrated biopsychosocial perspective in athlete development and rehabilitation. Addressing psychological factors systematically may enhance performance sustainability, reduce dropout, and improve long-term athlete well-being.

## Figures and Tables

**Table 2:** Characteristics of Included Studies Examining Psychological Determinants of Performance Slumps and Recovery (*n* = 14).

Author (Year)	Country	Sport/ Population	Sample (*n*)	Study Design	Psychological Variables	Outcome Variables	Follow-Up Duration	Key Finding
Beischer et al. (2019) [24]	Sweden	Adolescent and young adult athletes after ACL reconstruction	8 months: *n* = 384 12 months: *n* = 271	Case–control	Psychological outcome (ACL-RSI, Knee Self-Efficacy, Motivation)	Return to sport, muscle function recovery	8- and 12-month follow-up	Psychological readiness, self-efficacy and motivation associated with return-to-sport success
Boladeras et al. (2025) [34]	Spain	Elite female volleyball	*n* = 21	Longitudinal	POMS (Total Mood Disturbance), CSAI-2 (cognitive anxiety, somatic anxiety, self-confidence)	Injury incidence	Full season	Mood & anxiety predicted injury risk
Drew et al. (2025) [35]	Australia	Elite male League of Legends players (esports)	*n* = 5	Diary study (temporal)	Stress, coping	Coping effectiveness	Daily tracking	Stress intensity associated with threat appraisals; mastery coping was rated more effective.
Faleide et al. (2021) [29]	Norway	ACL reconstruction patients (Level I-III sports)	*n* = 129	Prospective cohort	Psychological readiness (ACL-RSI)	Return to preinjury sport	9 months predictor → 2-year outcome	Higher psychological readiness predicted successful return to preinjury sport
Garra et al. (2024) [31]	USA	ACL patients	*n* = 120	Matched cohort	Psychological readiness	RTS rates	Minimum 2 years	Psychological readiness is similar across groups
Madigan & Nicholls (2017) [9]	UK	Junior athletes	*n* = 93	Longitudinal	Mental toughness	Burnout	3-month follow-up (two-wave longitudinal)	Mental toughness reduced burnout development
Madigan et al. (2016) [8]	UK	Junior athletes	*n* = 141	3-wave longitudinal	Perfectionism, motivation	Burnout	6 months (three-wave longitudinal)	Motivation-mediated perfectionism-burnout link
McPherson et al. (2019) [25]	Australia	ACL athletes	*n* = 329	Prospective cohort	Psychological readiness	Second ACL injury	2 years	Lower readiness predicted reinjury
Mellano et al. (2022) [15]	USA	Collegiate female athletes	*n* = 126	Longitudinal	Coaching behaviors	Burnout	Two time-points across one competitive season	Negative coaching predicted burnout
Sanborn et al. (2022) [30]	USA	ACL patients	*n* = 100	Prospective RCT	Psychological readiness	RTS, ACL-RSI at 6, 12, 24 months	6, 12, and 24 months	Surgical technique influenced readiness
Slater et al. (2023) [44]	Australia	ACL-injured athletes treated non-surgically	*n* = 88	Prospective cohort	Psychological readiness (ACL-RSI), self-efficacy	Return to preinjury sport	3-, 6-, and 12-month follow-up	Psychological readiness independently associated with return to preinjury sport
Sorkkila et al. (2019) [20]	Finland	Student-athletes	*n* = 491	Longitudinal person-oriented	Resilience	Burnout & dropout	Multi-year	Resilience protected against burnout trajectory
Stenling et al. (2017) [14]	Sweden	Young elite skiers	*n* = 247	Longitudinal (latent growth)	Controlled motivation	Ill-being	Multi-wave	Controlling coaching increased ill-being
Zarzycki et al. (2024) [45]	USA	Female athletes after ACL reconstruction	*n* = 39	Prospective cohort	Psychological readiness (ACL-RSI), kinesiophobia (TSK-11)	Second ACL injury	2-year follow-up	Higher psychological readiness associated with increased second ACL injury risk

Abbreviations: ACL—Anterior Cruciate Ligament; ACL-RSI—Anterior Cruciate Ligament-Return to Sport after Injury Scale; RTS—Return to Sport.

**Table 3:** Risk of Bias Assessment of Included Studies (*n* = 14).

Author (Year)	Design	Selection Bias	Measurement Bias	Confounding Control	Attrition Bias	Reporting Bias	Overall Risk
Beischer et al. (2019) [24]	Case–control	Low	Low	Moderate	Low	Low	Low–Moderate
Boladeras et al. (2025) [34]	Longitudinal	Moderate	Moderate (self-report mood/anxiety)	Moderate	Low	Low	Moderate
Drew et al. (2025) [35]	Diary study (temporal)	High	Moderate (self-report stress)	Moderate	Low	Low	High
Faleide et al. (2021) [29]	Prospective cohort	Moderate	Low	Low	Moderate	Low	Low–Moderate
Garra et al. (2024) [31]	Matched cohort	Low	Low	Low (matched design)	Low	Low	Low
Madigan & Nicholls (2017) [9]	Longitudinal	Low	Moderate	Moderate	Low	Low	Low–Moderate
Madigan et al. (2016) [8]	3-wave longitudinal	Low	Moderate	Low	Moderate	Low	Low–Moderate
McPherson et al. (2019) [25]	Prospective cohort	Low	Low	Low	Low	Low	Low
Mellano et al. (2022) [15]	Longitudinal	Moderate	Moderate (burnout self-report)	Moderate	Low	Low	Moderate
Sanborn et al. (2022) [30]	Prospective RCT	Low	Low	Low	Low	Low	Low
Slater et al. (2023) [44]	Prospective cohort	Moderate	Moderate	Low	Moderate	Low	Moderate
Sorkkila et al. (2019) [20]	Longitudinal person-oriented	Low	Moderate	Low	Moderate	Low	Low–Moderate
Stenling et al. (2017) [14]	Longitudinal (latent growth)	Low	Moderate	Low	Moderate	Low	Low–Moderate
Zarzycki et al. (2024) [45]	Prospective cohort	Moderate	Low	Moderate	Low	Low	Low–Moderate

**Table 4:** Psychological Determinants and Performance Slump Outcomes.

Psychological Variable	Studies (*n*)	Outcome Type	Direction of Association	Strength of Evidence
Burnout	Madigan 2016 [8]; Madigan & Nicholls 2017 [9]; Mellano 2022 [15]; Sorkkila et al. [20]	Performance decline, dropout	↑ Burnout → ↑ Dropout; ↓ Well-being; ↓ Performance-related functioning	Strong (4 longitudinal)
Controlled Motivation	Stenling et al., 2017 [14]; Madigan 2016 [8]	Ill-being trajectory	↑ Controlled motivation → ↑ Ill-being	Moderate (single latent growth study)
Coaching Behaviors	Mellano 2022 [15]; Stenling et al., 2017 [14]	Burnout, ill-being	Controlling coaching → ↑ Burnout/↑ Ill-being	Moderate (2 longitudinal)
Stress (Affective/Cognitive)	Drew et al., 2025 [35]	Coping effectiveness	↑ Stress → ↑ Threat appraisal; ↓ Coping effectiveness	Weak–Moderate (*n* = 5 diary study)
Mood & Anxiety	Boladeras et al., 2025 [34]	Injury incidence	↑ Anxiety → ↑ Injury risk; Mood disturbance ↔ Anxiety (bidirectional)	Moderate (small sample longitudinal)
Mental Toughness	Madigan & Nicholls 2017 [9]	Burnout; performance	↑ Toughness → ↓ Burnout	Moderate (1 longitudinal)
Resilience	Sorkkila et al. [20]	Burnout; dropout risk	↑ Resilience → ↓ Burnout ↓ Dropout	Moderate–Strong (large multi-wave longitudinal)
Psychological Readiness (ACL-RSI)	Beischer et al. 2019 [24]; McPherson et al. 2019 [25]; Sanborn et al. 2022 [30]; Garra et al. 2024 [31]; Faleide et al. 2021 [29]; Slater et al. 2023 [44]; Zarzycki et al. 2024 [45]	Return-to-sport; reinjury	↑ Readiness → ↑ RTS; Reinjury findings inconsistent	Strong (RTS); Mixed (Reinjury)

Note: ↑ indicates increase; ↓ indicates decrease; → indicates a directional relationship between two variables; ↔ indicates bidirectional association.

**Table 5:** Integrated Synthesis of Psychological Determinants and Performance-Related Outcomes Across Included Studies.

Outcome Domain	Study (Author, Year)	Study Design	Psychological Determinant(s)	Direction of Effect	Strength of Evidence
Return to Sport (RTS)	Beischer et al. (2019) [24]	Case–control	ACL-RSI, Self-efficacy; Motivation	Higher readiness & self-efficacy → successful RTS	Moderate
	Faleide et al. (2021) [29]	Prospective cohort	ACL-RSI	Higher readiness → increased likelihood of return to preinjury sport	High
	Slater et al. (2022) [44]	Prospective cohort ACL-RSI; Self-efficacy Higher readiness independently predicted RTS at 12 months High			
	Sanborn et al. (2022) [30]	Randomized controlled trial	Psychological readiness (ACL-RSI)	Surgical method influenced readiness & RTS	Very High
	Garra et al. (2024) [31]	Matched cohort	Psychological readiness	No significant difference between fracture groups; readiness predicted RTS	Moderate–High
Reinjury Risk	McPherson et al. (2019) [25]	Prospective cohort	ACL-RSI	Lower readiness (particularly in younger athletes) → higher second ACL injury risk	High
	Zarzycki et al. (2023) [45]	Prospective cohort	ACL-RSI; Kinesiophobia	Higher readiness associated with increased second ACL injury risk	Moderate–High
Burnout/Performance Decline	Madigan et al. (2016) [8]	3-wave longitudinal	Perfectionism, motivation	Motivation mediated perfectionism → increased burnout	High
	Madigan & Nicholls (2017) [9]	2-wave longitudinal	Mental toughness	Higher toughness → reduced burnout over time	Moderate–High
	Mellano et al. (2022) [15]	Longitudinal	Controlling coaching behaviors	Controlling coaching → increased burnout across season	Moderate–High
	Sorkkila et al. (2019) [20]	Multi-year longitudinal	Resilience	Higher resilience → lower burnout trajectory & reduced dropout likelihood	High
	Stenling et al. (2017) [14]	Latent growth longitudinal	Controlled motivation; Controlling coaching	Controlled motivation → increased ill-being progression	High
Injury Incidence (Prospective)	Boladeras et al. (2025) [34]	Longitudinal (seasonal)	Mood disturbance; Cognitive anxiety	Higher cognitive anxiety & negative mood → increased injury risk	Moderate–High
Coping Effectiveness	Drew et al. (2025) [35]	Intensive diary study	Stress intensity; Coping strategies	Daily stress intensity → greater threat appraisal; mastery coping → higher coping effectiveness	Weak–Moderate

Note: → indicates a directional relationship between two variables.

## Data Availability

No new data were collected or examined in the course of this study. All pertinent information and original contributions are comprehensively documented within the manuscript itself. Consequently, the matter of data sharing does not apply to this work.

## Supplementary Materials

The following supporting information can be downloaded at: https://www.mdpi.com/article/10.3390/sports14050165/s1, File S1: PRISMA 2020 Checklist.
