# Supplementary material for: From Slump to Comeback: Psychological Determinants of Performance Decline, Burnout, and Recovery in Competitive Athletes—A Systematic Review
Source: Sports (Basel). 2026 Apr 22;14(5):165. doi: 10.3390/sports14050165 (PMC13211023; doi:10.3390/sports14050165)
Supplement: Supplementary file 1 [file sports-14-00165-s001.zip › sports-4215079-supplementary.pdf]

PRISMA 2020 Checklist

Section and Topic	Item #	Checklist item	Location where item is reported
**TITLE**			
Title	1	Identify the report as a systematic review.	Title page – “A Systematic Review”
**ABSTRACT**			
Abstract	2	Structured summary including background, objectives, methods, results, conclusions.	Abstract section (Page 1–2)
**INTRODUCTION**			
Rationale	3	Describe the rationale for the review in context of existing knowledge.	Introduction (Page 2–4)
Objectives	4	Provide explicit statement of objectives or research questions.	End of Introduction (Page 4–5)
**METHODS**			
Eligibility criteria	5	Specify inclusion/exclusion criteria and grouping of studies.	Section 2.1 Study Selection Procedures & Table 1 (Page 5–7)
Information sources	6	Specify databases, registers, and other sources searched.	Section 2.2 Literature Search (Page 5–6)
Search strategy	7	Present full search strategies including keywords and Boolean operators	Section 2.2 Literature Search (Page 6)
Selection process	8	Methods used to decide eligibility, number of reviewers, and screening process.	Section 2.1 Study Selection Procedures (Page 5)
Data collection process	9	Methods used to extract data and verify accuracy.	Section 2.3 Data Extraction (Page 6–7)
Data items	10a	Outcomes for which data were sought.	Section 2.3 Data Extraction (Page 6–7)
	10b	Other variables collected (sample size, design, instruments, etc.).	Section 2.3 Data Extraction (Page 6–7)
Study risk of bias assessment	11	Methods used to assess risk of bias in included studies.	Section 2.4 Methodological Quality (Page 7–8)

PRISMA 2020 Checklist

Section and Topic	Item #	Checklist item	Location where item is reported
Effect measures	12	Effect measures used (OR, regression coefficients, etc.).	Section 2.5 Summary Measures (Page 8)
Synthesis methods	13a	Process to decide study eligibility for synthesis.	Section 2.6 Synthesis of Results (Page 8)
	13b	Methods used to prepare data for synthesis.	Section 2.7 Data Synthesis (Page 8)
	13c	Methods used to present results (tables, diagrams)	Tables 2–5 (Page 9–14)
	13d	Methods used to synthesise results and rationale.	Section 2.7 Data Synthesis (Page 8)
	13e	Exploration of heterogeneity.	Section 2.9 Additional Analyses (Page 9)
	13f	Sensitivity analysis methods.	Section 2.9 Additional Analyses (Page 9)
Reporting bias assessment	14	Methods used to assess reporting bias.	Section 2.8 Publication Bias (Page 9)
Certainty assessment	15	Methods used to assess certainty of evidence.	Section 2.4 Methodological Quality (Page 7–8)
**RESULTS**			
Study selection	16a	Results of search and screening with flow diagram.	PRISMA Flow Diagram (Figure 1, Page 6)
	16b	Cite excluded studies and reasons.	PRISMA Flow Diagram & text (Page 6)
Study characteristics	17	Characteristics of included studies.	Table 2 (Page 9–11)
Risk of bias in studies	18	Risk of bias results.	Table 3 (Page 11–12)
Results of individual studies	19	Summary statistics and effect estimates.	Table 2 (Page 9–11)

PRISMA 2020 Checklist

Section and Topic	Item #	Checklist item	Location where item is reported
Results of syntheses	20a	Summary of characteristics and bias.	Results section (Page 9–12)
	20b	Results of synthesis.	Tables 4–5 (Page 12–14)
	20c	Investigation of heterogeneity.	Section 2.9 Additional Analyses (Page 9)
	20d	Sensitivity analyses.	Section 2.9 Additional Analyses (Page 9)
Reporting biases	21	Risk of bias due to missing results	Section 2.8 Publication Bias (Page 9)
Certainty of evidence	22	Overall certainty/confidence of evidence.	Results & methodological quality discussion (Page 11–14)
**DISCUSSION**			
Discussion	23a	Interpretation of results in context of evidence.	Discussion section (Page 15 onward)
	23b	Limitations of included evidence.	Discussion section
	23c	Limitations of review process.	Discussion section
	23d	Implications for practice and future research	Discussion section
**OTHER INFORMATION**			
Registration and protocol	24a	Provide registration information	Not registered (should be stated if applicable)
	24b	Protocol access.	Not specified
	24c	Amendments to protocol.	Not specified
Support	25	Sources of financial/non-financial support.	Acknowledgement / Funding section
Competing interests	26	Declare conflicts of interest.	Conflict of Interest statement

PRISMA

PRISMA 2020 Checklist

Section and Topic	Item #	Checklist item	Location where item is reported
Availability of data, code and other materials	27	Availability of data, code, and materials	Data availability statement

From: Page MJ, McKenzie JE, Bossuyt PM, Boutron I, Hoffmann TC, Mulrow CD, et al. The PRISMA 2020 statement: an updated guideline for reporting systematic reviews. BMJ 2021;372:n71. doi: 10.1136/bmj.n71. This work is licensed under CC BY 4.0. To view a copy of this license, visit https://creativecommons.org/licenses/by/4.0/
